# Host-Dependent Clustering of *Campylobacter* Strains From Small Mammals in Finland

**DOI:** 10.3389/fmicb.2020.621490

**Published:** 2021-01-13

**Authors:** Satu Olkkola, Mirko Rossi, Anniina Jaakkonen, Maria Simola, Jouni Tikkanen, Marjaana Hakkinen, Pirkko Tuominen, Otso Huitu, Jukka Niemimaa, Heikki Henttonen, Rauni Kivistö

**Affiliations:** ^1^Finnish Food Authority, Helsinki, Finland; ^2^Department of Food Hygiene and Environmental Health, Faculty of Veterinary Medicine, University of Helsinki, Helsinki, Finland; ^3^European Food Safety Authority (EFSA), Parma, Italy; ^4^Natural Resources Institute Finland (Luke), Helsinki, Finland

**Keywords:** *Campylobacter jejuni*, phylogeny, comparative genomics, rodent, mouse, vole, shrew

## Abstract

Small mammals are known to carry *Campylobacter* spp.; however, little is known about the genotypes and their role in human infections. We studied intestinal content from small wild mammals collected in their natural habitats in Finland in 2010–2017, and in close proximity to 40 pig or cattle farms in 2017. The animals were trapped using traditional Finnish metal snap traps. *Campylobacter* spp. were isolated from the intestinal content using direct plating on mCCDA. A total of 19% of the captured wild animals (*n* = 577) and 41% of the pooled farm samples (*n* = 227) were positive for *C. jejuni*, which was the only *Campylobacter* species identified. The highest prevalence occurred in yellow-necked mice (*Apodemus flavicollis*) and bank voles (*Myodes glareolus*) which carried *Campylobacter* spp. in 66.3 and 63.9% of the farm samples and 41.5 and 24.4% of individual animals trapped from natural habitats, respectively. Interestingly, all house mouse (*Mus musculus*) and shrew (*Sorex* spp.) samples were negative for *Campylobacter* spp. *C. jejuni* isolates (*n* = 145) were further characterized by whole-genome sequencing. Core genome multilocus sequence typing (cgMLST) clustering showed that mouse and vole strains were separated from the rest of the *C. jejuni* population (636 and 671 allelic differences, 94 and 99% of core loci, respectively). Very little or no alleles were shared with *C. jejuni* genomes described earlier from livestock or human isolates. FastANI results further indicated that *C. jejuni* strains from voles are likely to represent a new previously undescribed species or subspecies of *Campylobacter*. Core-genome phylogeny showed that there was no difference between isolates originating from the farm and wild captured animals. Instead, the phylogeny followed the host species-association. There was some evidence (one strain each) of livestock-associated *C. jejuni* occurring in a farm-caught *A. flavicollis* and a brown rat (*Rattus norvegicus*), indicating that although small mammals may not be the original reservoir of *Campylobacter* colonizing livestock, they may sporadically carry *C. jejuni* strains occurring mainly in livestock and be associated with disease in humans.

## Introduction

*Campylobacter* spp., especially *C. jejuni* and *C. coli*, are common causes of gastroenteritis in humans globally, campylobacteriosis being the most frequently reported zoonosis in the EU area (EFSA and ECDC, 2019). Many food-producing animal species are known to be asymptomatic carriers of campylobacters, and undercooked chicken is considered to be the main source for human disease, but also other livestock such as bovines have been associated with human cases (Cody et al., 2019; Joensen et al., 2020). However, the original reservoir and mechanisms of spread of these pathogens on livestock farms are not well understood.

Campylobacters are also known to commonly inhabit the digestive tracts of many other warm-blooded animals, including wild birds and small mammals (Meerburg et al., 2006; Backhans et al., 2013; Llarena et al., 2015; Kovanen et al., 2019). Rodents, among other potential hosts, have been suggested to spread *Campylobacter* spp. on farms (Meerburg and Kijlstra, 2007). Previous research has found a possible link between the presence of rodents, or the absence of control thereof, and higher *Campylobacter* prevalence in chicken flocks (Kapperud et al., 1993; Berndtson et al., 1996; Sommer et al., 2013; Agunos et al., 2014; Allain et al., 2014; Torralbo et al., 2014). Several rodent species such as the bank vole (*Myodes glareolus*), yellow-necked mouse (*Apodemus flavicollis*), house mouse (*Mus musculus*), and brown rat (*Rattus norvegicus*) have been shown to carry *Campylobacter* species in their intestinal tracts (Fernie and Healing, 1976; Healing and Greenwood, 1991; Meerburg et al., 2006; Backhans et al., 2013; Lõhmus and Albihn, 2013). Of these, in particular, *M. musculus* and *R. norvegicus* are species that commonly live in close proximity to human habitation, but also *M. glareolus* and *A. flavicollis* frequently invade human settlements at the onset of winter.

Little research exists on the prevalence and genotypes of *Campylobacter* spp. from small mammals in general and especially from those found on swine, beef or dairy farms. A study from Netherlands found approximately 10% of *M. musculus* and 12.5% (1/8) *R. norvegicus* from organic swine farms campylobacter-positive, but no shared genotypes with those from pig manure were detected with AFLP typing. No other rodent species or shrews were found to carry campylobacters (Meerburg et al., 2006). In contrast, a study from New Zealand found 11% of the rodents from a dairy farm to harbor campylobacters and identified several shared genotypes between rodent and cattle feces with pulsed-field gel electrophoresis (PFGE) (Adhikari et al., 2004). In Sweden, *C. jejuni* was more commonly detected from rodents captured in chicken farms while *C. coli* was more often found from rodents in pig farms, suggesting possible transmission from food-producing animals, but no genotyping was done to support this hypothesis (Backhans et al., 2013). Williams et al. (2010) studied *M. glareolus* and wood mice (*Apodemus sylvaticus*) for the presence of *Campylobacter* spp. both in woodland habitats and on cattle farms in the United Kingdom. They found 41 and 18% of *M. glareolus* from the woodlands and on farms to carry *Campylobacter* spp., respectively, while only 1% of the *A. sylvaticus*, and only on the farms, were positive. Furthermore, all isolates from *M. glareolus* represented a novel *C. jejuni* clone, multilocus sequence type (ST) 3704, which was also identified from a calf. This clone was also detected in three of the six positive *A. sylvaticus* while the other positive *A. sylvaticus* harbored isolates with ST 61 and ST 583, which were also identified from cattle.

While traditional multilocus sequence typing (MLST), based on seven house-keeping genes, has been widely used for the typing of *Campylobacter* isolates from various sources, more powerful typing methods based on whole-genome sequencing (WGS) give us considerably higher discriminatory power for phylogenetic analyses and epidemiologic and source attribution studies (Llarena et al., 2017). Also, antimicrobial resistance phenotypes can be predicted based on WGS data in *Campylobacter* species (Zhao et al., 2015). Only a few studies exist worldwide that utilize new methods such as WGS-based typing on *Campylobacter* isolates from small wild mammals (Kim et al., 2020). Their role as a source of *Campylobacter* spp. for food-producing animals in Finland has not been explored previously.

Our aims were (i) to explore the occurrence, genotypes, and resistance markers of *Campylobacter* spp. isolates from small mammals, mainly rodents and shrews, caught from 40 Finnish beef, dairy, and swine farms, and from natural habitats throughout Finland, (ii) to compare them to isolates collected from other sources, and (iii) to estimate their role as vectors for *Campylobacter* spp. for Finnish livestock and humans.

## Materials and Methods

### Sampling

During October and November 2017, rodent traps were set on 40 farms in western and south-western Finland. Of these, 18 were beef cattle farms, two were dairy farms and 20 were swine farms. Both live and snap traps (traps that instantly kill the rodent) – 100 traps in total – were set at and near production buildings and at the edges of the farmyard areas. The small mammals (*n* = 442) were caught by Natural Resources Institute Finland. The trapping was done over two consecutive nights at every farm and the traps were checked in each day. These were all included as farm samples in the successive analyses.

Additionally, small mammals were collected using snap traps in the national regulatory monitoring of the vole population by the Natural Resources Institute Finland in June 2015 (*n* = 128), and in May–June and September–November 2017 (*n* = 384). The trapping sites were located throughout Finland in forest and field habitats (Korpela et al., 2013). These constituted the samples from the wild.

A sample (*n* = 65) of *A. flavicollis* trapped inside office and storage buildings in southern Finland in 2010–2015 was also included for comparison.

### Sample Preparation and Isolation of *Campylobacter* spp.

Most of the animals, 86%, were dissected on the day they were trapped. The whole intestines were frozen in dry ice for transport and stored at −80°C in the laboratory. Samples were thawed at room temperature for 1–2 h before starting analysis. For practical reasons, a portion of the animals, 14%, were first stored in dry ice in the field, and then at −80°C as whole animals. After thawing, the intestines were dissected in a similar way to the freshly prepared animals and analyzed immediately.

Sample preparation was performed following the ISO-6887/6 (2013): The intestines and stomachs were first cut into small pieces with a sterile scalpel. Then, the subsamples were pooled so that one pool contained subsamples from a maximum of ten individuals of the same species, caught from a single farm on the same night. After pooling, 1:1 (w/w) buffered peptone water was added to the pooled sample. The sample was cultivated following the ISO-10272-1 (2017) standard Detection procedure C. The colonies were confirmed as *C. jejuni* using MALDI-TOF (Maldi^®^ Biotyper, Bruker Daltonics GmbH, Germany, reference library version 8.0.0.0) with cut-off at 2.0. The colonies identified as *C. jejuni* with a score lower than 2.0 were further confirmed with microscopy. After identification, the isolates were stored in media containing brain heart infusion broth and 15% glycerol at −80°C.

Similarly, the intestines from individual animals trapped in their natural habitats were homogenized using a cotton swab dipped in sterile buffered peptone water and subsequently plated on mCCDA plates incubated under microaerobic conditions (5% ± 2% O_2_, 10% ± 3% CO_2_, ≤10% H_2_, balanced with N_2_; Anoxomat System, Mart Microbiology, Netherlands or ThermoForma Series 2 Water Jacketed Incubator (HEPA filter) Model 3131, Thermo Fisher Scientific, United States) at 41.5 ± 1.0°C for 48–72 h. One typical colony was confirmed as *C. jejuni* by the lack of aerobic growth on blood agar at 25°C, microscopy, and using species-specific PCR (Denis et al., 1999).

### Bacterial Strains, DNA Extraction, and Whole-Genome Sequencing

A selection of *C. jejuni* isolates from the farm samples (see below) and all 102 isolates from the wild animal samples were subjected to WGS (of which 99 were included in the cgMLST, phylogenetic, ANI, and AMR analyses (Supplementary Datasets), and two additional strains were included only in the MLST and AMR analyses (Table 1); one strain failed at the sequence assembly and QC stage. Of the farm samples, one isolate per farm and per animal species was selected, giving a total of 46 strains. Genomic DNA was extracted with PureLink Genomic DNA kit (Thermo Fisher Scientific, United States) or DNeasy Blood and Tissue kit (Qiagen, United States), according to the manufacturer’s instructions. The purity of DNA samples was tested using NanoDrop-apparatus (Thermo Fisher Scientific, United States), accepting the extraction if the A260/280-ratio exceeded 1.8 and the A260/230 ratio exceeded 2.0. DNA concentration was measured using Qubit-fluorometer (Thermo Fisher Scientific, United States) and Qubit dsDNA Broad Range -kit (Thermo Fisher Scientific, United States). The extracted DNA was stored at −20°C.

**TABLE 1:** Prevalence of *Campylobacter jejuni* in different animal species and sampling habitats, and sequence types (STs) detected.

Common name	Scientific name	No. of pooled samples from farms in 2017 (%-positive)	No. of animals from natural habitats in 2015 and 2017 (%-positive)	STs detected (no. of isolates)^*b*^
Yellow-necked mouse	*Apodemus flavicollis*	89 (66.3%)	1 (0%), 65^*a*^(41.5%)	1,304 (1 + 0), 2,219 (1 + 0), **9,468** (1 + 0), **9,471** (1 + 0), **9,477** (19 + 22)
House mouse	*Mus musculus*	36 (0%)	0 (0%)	–
Harvest mouse	*Micromys minutus*	10 (70.0%)	2 (50.0%)	**9,470** (2 + 1), **9,474** (3 + 0)
Brown rat	*Rattus norvegicus*	10 (20.0%)	0 (0%)	4,791 (1), **9,475** (1)
Bank vole	*Myodes glareolus*	36 (63.9%)	266 (24.4%)	1,304 (2 + 0), 3,704 (0 + 1), 8,562 (0 + 1), **9,467** (0 + 2), **9,469** (1 + 2), **9,472** (2 + 3), **9,473** (6 + 38), **9,476** (3 + 0), **9,825** (0 + 7), **9,826** (0 + 1), **9,827** (0 + 1), **9,828** (0 + 1), **9,829** (0 + 2), **9,858** (0 + 2), **9,859** (0 + 2)
Red vole	*Myodes rutilus*	0 (0%)	11 (63.6%)	**9,473** (1), **9,859** (3), **9,860** (1), **9,861** (2)
Gray-sided vole	*Craseomys* (*Myodes*) *rufocanus*	0 (0%)	17 (0%)	–
East European vole	*Microtus mystacinus* (*M. levis*)	12 (8.3%)	1 (0%)	**9,476** (1)
Field vole	*Microtus agrestis*	4 (25.0%)	65 (12.3%)	**9,467** (1 + 1), **9,472** (0 + 1), **9,473** (0 + 5), **9,825** (0 + 1)
Tundra/root vole	*Alexandromys* (*Microtus*) *oeconomus*	0 (0%)	55 (0%)	–
Wood lemming	*Myopus schisticolor*	0 (0%)	5 (0%)	–
Common shrew	*Sorex araneus*	23 (0%)	82 (0%)	–
Taiga shrew	*Sorex isodon*	2 (0%)	0 (0%)	–
Pygmy shrew	*Sorex minutus*	2 (0%)	4 (0%)	–
Laxmann’s shrew	*Sorex caecutiens*	0 (0%)	1 (0%)	–
Water shrew	*Neomys fodiens*	1 (0%)	2 (0%)	–
Least weasel	*Mustela nivalis*	2 (0%)	0 (0%)	–
**Total**		**227 (41.0%)**	**577 (18.7%)**	

*^a^Apodemus flavicollis samples collected in 2010–2015 from buildings (other than on a farm) or their surroundings. ^b^In case C. jejuni was isolated from both sampling habitats, number of isolates from each habitat is indicated in parenthesis (from farms + natural habitats). Farms were located in southern or western Finland, while samples from natural habitats were more dispersed throughout Finland in 2017 (only northern Finland was included in 2015). STs first described in this study are indicated in bold. Synonyms in parentheses.*

WGS of the farm isolates was outsourced to a commercial company (CeGat GmbH, Tuebingen, Germany). The sequencing of the wild animal isolates was performed by Istituto Zooprofilattico Sperimentale Dell’Abruzzo e Molise, Teramo, Italy under a Memorandum of Intent between the faculty and the institution. Sequencing libraries were prepared with a Nextera XT Library Prep kit (Illumina, San Diego, CA, United States) and sequenced on the Illumina NovaSeq6000 or NextSeq platform (Illumina, United States), with a 2 × 100-bp paired-ended protocol according to the manufacturer’s instructions, aiming at >100X coverage.

### Assembly, Multilocus Sequence Typing, and Core Genome MLST Analysis

Draft genome sequences were assembled using a docker image of INNUca v3.1 (Machado et al., 2017) available at^1^. In brief, after calculating if the sample raw data fulfils the expected coverage (min. 15x), INNUca checks read quality using FastQC^2^, and processes the reads using Trimmomatic (Bolger et al., 2014). Then, INNUca proceeds to *de novo* draft genome assembly with SPAdes 3.11 (Bankevich et al., 2012), followed by checking the assembly depth of coverage (min. 30x) and improving assembly using Pilon (Walker et al., 2014). Finally, 7-gene MLST sequence type (ST) is automatically assigned with the mlst software (Seemann, 2017). This analysis made use of the PubMLST website^3^ developed by Keith Jolley (Jolley and Maiden, 2010), sited at the University of Oxford. The development of this website was funded by the Wellcome Trust. Assembly statistics are available in Supplementary Dataset 1.

Core genome MLST analysis was performed using a docker image of the chewBBACA suite (Silva et al., 2018) available at^4^ and the INNUENDO cgMLST schema available in Zenodo^5^ (Rossi et al., 2018), consisting of 678 loci. In addition to 145 sequenced *C. jejuni* genomes from rodents and shrews, the cgMLST analysis included 6,526 genomes belonging to the reference INNUENDO dataset (Rossi et al., 2018) and other genomes previously sequenced (Kovanen et al., 2019), 6,830 genomes in total. The genomes had no more than 2% of missing loci. Metadata, including year of isolation, country, 7-gene MLST ST, and source for all the strains, are available in Supplementary Dataset 2. The allelic profiles were clustered using the globally optimal eBURST (goeBURST) algorithm (Francisco et al., 2009) in PHYLOViZ V 2.0 software (Francisco et al., 2012), and clusters were generated at all possible thresholds.

### Allele Segregation

To analyze allele segregation according to source, the cgMLST profiles of a total of 4,323 strains for which source information was available were analyzed using proCompare.py (Pinho et al., 2016) available at^6^. Briefly, the software performs a pairwise comparison searching those loci with shared (or exclusive) alleles between two groups.

### Average Nucleotide Identity Calculations

To evaluate the taxonomical position of the newly described *C. jejuni* genomes, all-against-all pairwise average nucleotide identity (ANI) values for the mouse and vole strains were calculated using FastANI (v1.0) with default parameters (Jain et al., 2018). In addition, ANI values were calculated between each strain and the following reference genomes: *C. jejuni* subsp. *jejuni* NCTC 11168 (GenBank Accession number: NC_002163.1), *C. jejuni* subsp. *jejuni* RM1221 (NC_003912.7), *C. jejuni* subsp. *doylei* 269.97 (CP000768.1), *C. coli* 76339 (NC_022132.1), and *C. coli* OR12 (NZ_CP019977.1).

### Phylogenomics

A phylogenetic tree was constructed based on the core genomes of 1,406 *C. jejuni* strains selected among the 6,830 genomes, as described above, excluding the samples isolated from humans and those without information on source (Supplementary Dataset 2). The genomes were annotated using a docker image of prokka 1.12 (Seemann, 2014) available at^7^, and pan-genome analysis was performed using a docker image of Roary 3.7.0 with default settings (Page et al., 2015) available at^8^. The jobs were run in parallel using GNU Parallel (Tange, 2011). FastTree 2.153 (Price et al., 2009, 2010) was used with the Jukes-Cantor model of nucleotide evolution for building the approximation of a maximum-likelihood (ML) phylogenetic tree based on the core genome alignment (99% shared loci in Roary analysis, including 864 core genes). iTOL54 v4.2.3 (Letunic and Bork, 2019) was used for visualization. The tree was rooted at midpoint.

For improving the resolution of the phylogeny of the vole and mice specific lineages, *ad hoc* pangenome analysis was performed. The resulting nucleotide multiple core genome alignment files were used as an input for phylogenetic inference using maximum likelihood methodology implemented in iqtree (Nguyen et al., 2015), applying model selection (Kalyaanamoorthy et al., 2017) and ultrafast bootstrapping (Hoang et al., 2018). The core-genome alignments and the ML trees generated by iqtree were used for the subsequent assessment of recombination using ClonalFrameML (Didelot and Wilson, 2015) ignoring sites with any ambiguous bases. The trees were rooted at midpoint.

### Antimicrobial Resistance

The antimicrobial resistance determinants of the isolates were searched from the quality-controlled assemblies using default settings in ResFinder 3.2 (Zankari et al., 2012), accessed April 24th and 25th, 2020. The resistance gene sequences were aligned using Clustal Omega^9^ and blasted in the NCBI Blast nucleotide^10^ against the nucleotide collection (nr/nt) (retrieved September 25th, 2020).

### Statistical Analysis

Statistical significance of the observed differences in *Campylobacter* prevalence between different sampling times and locations were tested using the Chi-square or Fisher’s Exact tests in Microsoft Excel 2016. Statistically significant differences were considered for *p*-values below 0.05.

## Results

*A. flavicollis*, *M. musculus*, *Micromys minutus* (harvest mouse), and *R. norvegicus* were almost exclusively caught on-farm (year 2017) or inside buildings (years 2010–2015, *A. flavicollis* collection) (Table 1). The distribution of red voles (*Myodes rutilus*), tundra/root voles (*Alexandromys oeconomus*), and gray-sided voles (*Craseomys rufocanus*) are limited to northern parts of Finland and accordingly, these species were not caught on farms in south-western or western Finland. *M. glareolus*, *Sorex araneus* (common shrew), and *Microtus agrestis* (field vole) were common among both in-farm caught samples and those collected from natural habitats throughout Finland.

### Prevalence of *Campylobacter* spp. Was Highest in *A. flavicollis*

A total of 41% (93/227) of the pooled samples from farms, and 19% (75/384 in 2017, 6/128 in 2015, and 27/65 for *A. flavicollis*) of the individual animals captured from office and storage buildings were positive for *C. jejuni* (Table 1), which was the only *Campylobacter* species identified. The highest prevalence of *C. jejuni* occurred in *A. flavicollis*, *M. minutus*, and *M. glareolus*, *M. rutilus*, and *M. agrestis*. Interestingly, all shrew, *Alexandromys (Microtus) oeconomus* (tundra/root vole), and *M. musculus* samples were negative for *Campylobacter* species.

*M. glareolus* from natural habitats was more likely to carry *Campylobacter* when caught from fields (60%) compared to forests (18%) (*p* < 0.0001) in 2017. The samples from fields were comparable in *Campylobacter* prevalence with those from farms. In natural habitats, *M. glareolus* was significantly more often *Campylobacter-*positive in southern Finland (42%) compared to Lapland (19%) in northern Finland (*p* < 0.0001) in 2017. In Lapland, *Campylobacter* prevalence in *M. glareolus* was significantly higher in 2017 (19%) compared to 2015 (7%) (*p* = 0.0001). For other parts of the country (excluding Lapland), we had samples only from 2017.

### MLST Analysis Revealed Genotypes Differing From Livestock and Humans

Only seven out of the 147 *C. jejuni* isolates from mice, rats, or voles matched a previously defined 7-gene MLST profile, most of which were associated with a clonal complex (CC): ST 1304 (*n* = 3, ST-1304 CC), ST 2219 (*n* = 1, ST-45 CC), ST 3704 (*n* = 1), ST 4791 (*n* = 1, ST-45 CC), or ST 8562 (*n* = 1) (Supplementary Dataset 1). For the rest of the dataset, new alleles or new combinations of alleles were detected (Table 1). All the isolates with STs belonging to ST-45 CC or ST-1304 CC (*n* = 5) were from rodents captured from farms. ST-45 CC was isolated from *A. flavicollis* from a pig farm (ST 2219) and from *R. norvegicus* from a cattle farm (ST 4791). ST 1304 was isolated from *A. flavicollis* from a cattle farm and from two *M. glareolus* from pig and cattle farms. Two other previously identified STs, ST 3704 and ST 8562, were both isolated from *M. glareolus* from a forest in central Finland and from northern Finland, respectively.

Novel STs that were most prominent in the dataset included ST 9473 from *M. glareolus*, *M. agrestis* and *M. rutilus*, and ST 9477 only identified in *A. flavicollis*. ST 9473 was identified among *C. jejuni* isolates from *M. glareolus* from both on-farm (pig and cattle farms) and natural habitats in both 2015 and 2017. ST 9477 was identified among *C. jejuni* isolates from *A. flavicollis* from both on-farm (pig and cattle farms) and other buildings (office and storage buildings) in southern Finland from 2010 to 2017. In addition, ST 9470 was isolated from *M. minutus* and ST 9467 from *M. agrestis* caught both on-farm and from natural habitats. Furthermore, the same STs occurred in *C. jejuni* isolates from both *M. agrestis* and *M. glareolus*.

### cgMLST and Allele Segregation Analyses Further Highlighted the Differences

Of the 138 *C. jejuni* strains isolated from voles and mice with previously undefined ST designation, the cgMLST based on 678 loci yielded a total of 105 unique profiles. This revealed that the *C. jejuni* strains isolated from these animals form distinct populations that diverge largely from the ones described so far. Changes in the composition of clusters was investigated at all goeBURST thresholds. At the threshold of 671 allelic differences (99.0% of the core loci), the *C. jejuni* strains from voles formed two separate clusters from the rest of the strains. At 636 allelic differences (93.8% of core genes), the *C. jejuni* strains from mice separated from the larger cluster (composed by strains isolated from livestock, humans, and wild birds), which was split further into two sub-clusters at 531 allelic differences (78.3% of core genes). One contained strains isolated exclusively from *A. flavicollis* and one was composed of strains isolated exclusively from *M. minutus* (Supplementary Dataset 3). Descriptive statistics of the pairwise distance among strains of each group are summarized in Table 2.

**TABLE 2:** Pairwise allelic distance within each goeBURST group defined at threshold 531 allelic differences based on core genome multilocus sequence typing (cgMLST) schema composed by 678 loci.

goeBURST group	Source	Taxa	Average	Median	Max	SD
7	Voles	*Microtus agrestis*, *Microtus mystacinus*, *Myodes rutilus*, *Myodes glareolus*	280.7	332	410	111,0
21	Voles	*Myodes rutilus*, *Myodes glareolus*	159.2	164	300	102.7
13	Mice	*Apodemus flavicollis*	78.1	84	147	34.8
27	Mice	*Micromys minutus*	128.2	134	251	115.2

Considering the large diversity in the cgMLST profiles observed between mouse and vole strains and the rest of the *C. jejuni* population, we investigated how many core loci alleles were shared between *C. jejuni* isolates obtained from different taxa. To perform this study, we searched how many times an allele detected in a *C. jejuni* strain isolated from one mouse or vole taxon was also detected in a *C. jejuni* strain isolated from another taxon. For this analysis, seven strains isolated from mice and voles but not belonging to the four goeBURST groups described above were excluded. Supplementary Dataset 4 shows the results of the pairwise comparison. The *C. jejuni* isolates obtained from *A. flavicollis* shared 184 loci with strains isolated from *M. minutus* and a median of 2 (max 6) and 53 (max 141) with voles and other taxa, respectively. Similar results were obtained for strains isolated from *M. minutus.* Very few alleles were shared between strains isolated from voles and any other taxa, 18 being the maximum number of shared alleles detected. Overall, this analysis confirmed the divergent nature of the *C. jejuni* population circulating both in mice and voles.

### FastANI and Phylogenetic Analyses Revealed That Vole Isolates Probably Form a Novel (Sub-)Species of *Campylobacter*

**FIGURE 1:**
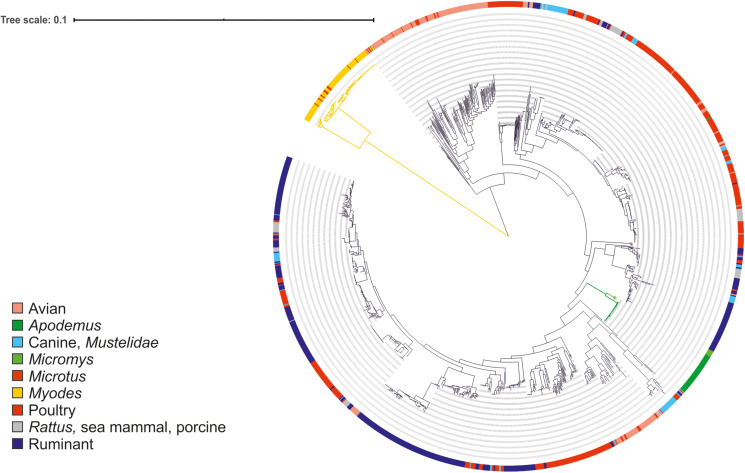
Maximum likelihood phylogenetic tree based on the alignment of 864 core genes of 1,406 *C. jejuni* strains from various sources. The tree was rooted at mid-point. The clade associated with voles is shown in yellow and the clades associated with mice in green. Color in the external ring indicates the source of the strain (see legend).

The little or no sharing of alleles between the *C. jejuni* isolated from voles and other taxa is an indication that these strains might form a different *Campylobacter* species or subspecies. To verify this hypothesis, we calculated the ANI percentage between mouse and vole strains and reference strains from *C. jejuni* subspecies and *C. coli* (Table 3). Compared with the reference genomes of *C. jejuni* and *C. coli*, the ANI values calculated for vole strains were significantly lower than the ones calculated for the mouse strains (unpaired *t*-test; *P* < 0.001). The mouse strains had >95% ANI versus *C. jejuni* subspecies and <87% ANI versus *C. coli*. On the contrary, the vole strains had on average 90.9% ANI versus *C. jejuni* subspecies and <84% ANI versus *C. coli*. Both *Campylobacter* strains from voles and mice formed cohesive groups with ANI average at 99% and min. 96%. This data confirmed the hypothesis that *Campylobacter* strains from voles might form a different taxonomic group. The maximum likelihood phylogenetic tree based on the alignment of 864 core genes supports the ANI results (Figure 1). The vole strains formed a distinct clade clearly separated from *C. jejuni*, while the strains isolated from mice grouped monophyletically within the diversity of the *C. jejuni* population.

**TABLE 3:** All-versus-all average nucleotide identity (ANI%).

Group	Reference	Average	Median	Min	Max
Voles	Voles	**99.17**	**99.66**	**96.48**	**100.00**
	Mice	91.33	91.38	90.76	91.61
	*C. jejuni* subsp. *jejuni*	91.06	91.06	90.82	91.26
	*C. jejuni* subsp. *doylei*	90.67	90.66	90.53	90.81
	*C. coli*	83.45	83.47	82.59	84.45
Mice	Mice	**99.14**	**99.93**	**95.75**	**100.00**
	Voles	91.28	91.32	90.69	91.56
	*C. jejuni* subsp. *jejuni*	**97.57**	**97.60**	**96.73**	**97.74**
	*C. jejuni* subsp. *doylei*	**95.59**	**95.60**	**95.22**	**95.73**
	*C. coli*	84.50	84.70	83.06	86.80

*In bold values > 95%.*

For investigating the presence of phylogeographical or temporal signals and to see whether there were differences between strains isolated from animals captured in the proximity of the farm or not, we reconstructed the genealogy of the mouse and vole specific lineages based on *ad hoc* core-genome alignment. The phylogenetic reconstructions were based on a core genome alignment of 1,238 and 1,402 genes for the vole and the mouse lineages, respectively. For the vole lineages, ClonalFrameML detected a total of 1,059 recombination events, 337 on terminal branches and the rest on internal nodes, and the imported sizes ranged between 2 and 103,802 bp (median 419). For the mouse lineages, 225 recombination events were detected, 32 on terminal branches and the rest on internal nodes, and the imported sizes ranged between 2 and 6,597 bp (median 712). The posterior mean calculated for the ratio of recombination events compared to mutation (rho/theta) was 0.342058 (posterior variance 1.82762 × 10^–05^) for the vole and 0.111041 (posterior variance 2.78479 × 10^–05^) for the mice. This analysis showed that mutation was approximately 3 and 9 times more frequent than recombination in the vole and mouse lineages, respectively.

### Geographical and Temporal Differences Between Genotypes Were Small

**FIGURE 2:**
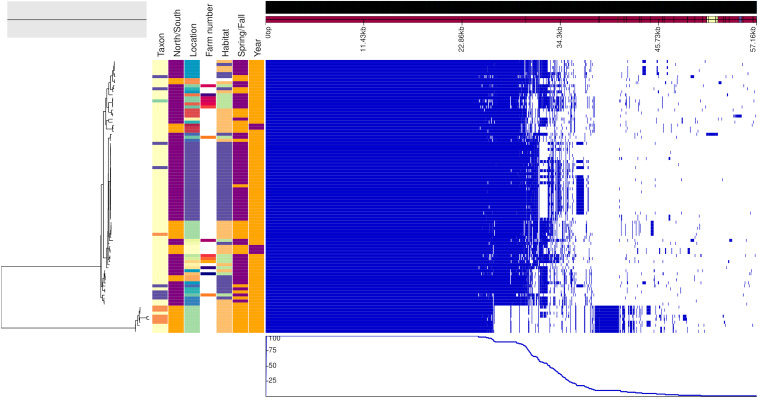
Genealogy of vole-associated *Campylobacter* lineage (left) with associated metadata, i.e., taxon (*M. glareolus* in light yellow, *M. rutilus* in orange, *M. agrestis* in violet, and *M. mystacinus* in turquoise), geographical area (north Finland in orange and south in purple), location, farm number (if applicable), habitat (field in blue, forest in orange, and farm in green), time (spring in orange and fall in purple) and year (2015 in purple and 2017 in orange), displayed alongside the gene presence (blue)/absence (white) plot from the Roary pangenome analysis. The figure was drawn using the phandango.net web application (Hadfield et al., 2017). The phylogeny based on 1,238 core genes was reconstructed using ClonalFrameML (Didelot and Wilson, 2015) and rooted at mid-point.

**FIGURE 3:**
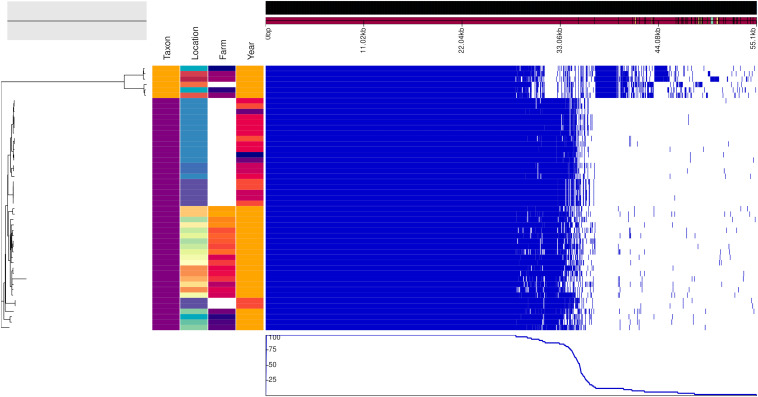
Genealogy of mice-associated *Campylobacter* lineage (left) with associated metadata, i.e., taxon (*A. flavicollis* in purple and *M. minutus* in orange), location, farm (if applicable) and year (2017 in orange, 2015 in dark orange, 2014 in light red, 2013 in dark red, 2011 in violet, and 2010 in blue), displayed alongside the gene presence (blue)/absence (white) plot from the Roary pangenome analysis. The figure was drawn using the phandango.net web application (Hadfield et al., 2017). The phylogeny based on 1,402 core genes was reconstructed using ClonalFrameML (Didelot and Wilson, 2015) and rooted at mid-point.

Figures 2, 3 show the genealogies of the vole and mouse lineages, respectively, alongside the information concerning the site and time of capture of the animals. The *Campylobacter* strains did not segregate clearly according to space or time, and no major differences were observed between animals captured within farms and those captured in the wild. However, the smaller cluster of *Campylobacter* strains from voles, which diverged significantly from the main population (Figure 2), consisted of samples collected only from one location in Lapland and represented all except one isolate from *M. rutilus* (orange taxon) and few isolates from *M. glareolus* (yellow taxon). Furthermore, for the *Campylobacter* isolates from mice, the smaller cluster consisted of strains from *M. minutus* (orange taxon) and the larger one from *A. flavicollis* (purple taxon) (Figure 3). The *Campylobacter* strains from *A. flavicollis* showed some minor clustering according to location.

### Antimicrobial Resistance Markers

We found a nucleotide substitution resulting in P104S amino acid change in GyrA in 62.6% (92/147) of the *C. jejuni* isolates (Table 4). No other known resistance-associated mutations were identified using Resfinder 3.2. The remaining isolates apart from one, 36.7% (54/147) harbored a beta-lactamase gene, the type of which was associated with the ST. Of these, 79.6% (43/54) had a nucleotide (T) deletion at position 69 of the betalactamase gene resulting in a frameshift and premature stop codon at amino acid 35 and most likely a non-functional gene product (all from *A. flavicollis*, mainly ST-9477). Only one isolate had a beta-lactamase gene with 100% nucleotide identity to that previously deposited to Genbank. Not surprisingly, this isolate, derived from *A. flavicollis* trapped on a swine farm, had a previously recognized ST (ST 2219, ST-45 CC). All isolates that had the same ST carried identical resistance markers. Both the *gyrA* mutation and beta-lactamase genes were never present in the same isolates, and only one isolate [from *R. norvegicus* trapped from a cattle farm having ST 4791 (ST-45 CC)] had neither.

**TABLE 4:** Detected resistance markers.

Betalactamase gene (accession)^1^	% identity	% coverage	Known mutations	n/n all (%)	Species	MLST (*n* isolates with marker present/n all isolates with ST)
–	–	–	GyrA: P104S	92/147 (62.5%)	*Microtus agrestis, Myodes glareolus, Microtus mystacinus, Rattus norvegicus, Myodes rutilus*	3,704 (1/1), 8,562 (1/1), 9,467 (4/4), 9,469 (3/3), 9,472 (6/6), 9,473 (50/50), 9,476 (4/4), 9,825 (8/8), 9,826 (1/1), 9,827 (1/1), 9,828 (1/1), 9,829 (2/2), 9,858 (2/2), 9,859 (5/5), 9,860 (1/1), and 9,861 (2/2)
blaOXA-184 (NG_049485.1)	99.87^2^	100	–	43/147 (29.3%)	*Apodemus flavicollis*	9,468 (1/1), 9,471 (1/1), and 9,477 (41/41)
blaOXA-627 (NG_057498.1)	99.87	100	–	6/147 (4%)	*Micromys minutus*	9,474 (3/3) and 9,470 (3/3)
blaOXA-447 (NG_049732.1)	100	100	–	1/147 (0.7%)	*Apodemus flavicollis*	2,219 (1/1)
blaOXA-617 (NG_057550.1)	99.61	100	–	3/147 (2%)	*Myodes glareolus, Apodemus flavicollis*	1,304 (3/3)
blaOXA-448 (NG_049733.1)	98.39	100	–	1/147 (0.7%)	*Rattus norvegicus*	9,475 (1/1)

*^1^Closest hit from GeneBank using blast nucleotide. ^2^Truncated due to nucleotide deletion and frameshift.*

## Discussion

### Prevalence of *C. jejuni* in Rodents and Shrews

*Campylobacter jejuni* occurred in nearly all animal species sampled, even those collected from the less populated northern parts of Finland (including red, tundra, and gray-sided voles). The prevalence of *Campylobacter* was slightly higher among animals trapped on farms compared to those caught in their natural habitats. However, this is likely due to the fact that the farm samples were pooled from 1–10 individual animals compared to the animals from natural habitats which were studied individually. *A. flavicollis* were almost exclusively (except for one) caught near or inside buildings and mainly in the fall, and showed the highest prevalence (42% for individual animals trapped from natural habitats, 66% for pooled farm samples) of *Campylobacter*. Previously, *C. jejuni* has rarely been isolated from *A. flavicollis* (1/45, 2%), from human dwellings during the cold season in rural areas around Uppsala, Sweden (Lõhmus and Albihn, 2013). In another study from Sweden, *C. jejuni* was isolated from 5/18 (28%) and *C. upsaliensis* from 2/18 (11%) *A. flavicollis*, respectively, from pig farms, chicken farms, and non-farm locations in Sweden (Backhans et al., 2013), showing more comparable results with our study, which included a total of 155 samples from this species. However, all the isolates from our study were identified as *C. jejuni*. In this study, no *Campylobacter* spp. were detected in *A. flavicollis* caught in one location for three successive years. Among the other locations and years studied, the prevalence of *Campylobacter* ranged from 20 to 86%.

*M. glareolus*, which was common among both samples from farms and from natural habitats, also showed a high prevalence of *Campylobacter* (24% of individuals, 64% of pooled farm samples). Another common mammal that is widespread in Europe, *M. agrestis*, was less often (12% of individuals, 25% from pooled farm samples) positive for *Campylobacter* than *M. glareolus*. Previous studies have reported contradictory results concerning the occurrence of campylobacter in *M. glareolus*. While research from the United Kingdom and Sweden showed a prevalence of 18–77% (Fernie and Healing, 1976; Williams et al., 2010; Lõhmus and Albihn, 2013), a study from Norway (Rosef et al., 1983) found no positive animals, suggesting possible geographical differences in the occurrence, even though methodological variation might also have influenced the outcomes. Furthermore, *Campylobacter* spp. have not been isolated from *M. agrestis* either in the United Kingdom or in Sweden (Fernie and Healing, 1976; Meerburg et al., 2006), which also suggests geographical differences.

In Lapland (Pallasjärvi, Muonio), *M. glareolus* had a clearly higher prevalence in 2017 than 2015. A continuous long-term monitoring of vole dynamics has been running in this collection area since 1970 (Henttonen et al., 1987; Henttonen, 2000). Consequently, we can compare the campylobacter infection parameters with the vole densities. The spring–fall density indices (animals per 100 trap nights) of *M. glareolus* during 2013–2018 were: 2013: 0.3–5.1; 2014: 5.0–25.3; 2015: 11.1–31.3; 2016: 12.6–23.6; 2017: 5.2–3.8; 2018: 0.1–4.4. Thus, sampling in 2015 was in the middle of an extended high-density period while in 2017 it was in the decline–low phase. Prevalence was not directly density-dependent, rather there seemed to be a delayed density-dependent pattern. Parasitological parameters in small mammals depend largely on the population structure of the sample (sex, age structure, breeding or not, etc.) and we emphasize that this comparison was made between similar samples, i.e., breeding bank voles in early summer. Even if the exact reason for the great difference between 2 years is not known, it is important to realize generally in epidemiological sampling that temporal differences can be pronounced.

*M. musculus* and *S. araneus* were consistently *Campylobacter* negative despite being quite common among the studied samples. A similar finding concerning *M. musculus* was presented recently from South Korea (Kim et al., 2020) involving 49 *M. musculus* trapped on sesame fields. In another study conducted on pig farms, chicken farms, and at non-farm locations in Sweden, however, *C. jejuni* was isolated from 2%, *C. coli* from 12% and *C. upsaliensis* from 2% of the *M. musculus* samples, respectively (Backhans et al., 2013). The authors concluded that *C. jejuni* was more common on chicken farms and *C. coli* on pig farms, suggesting that rodents are not the original source of *Campylobacter* on farms but rather become carriers through contact with the feces of farm animals. However, in France *C. jejuni*, instead of *C. coli*, was identified from 12% of the *M. musculus* batches on pig farms (LeMoine et al., 1987). In another study, one (7%) *C. hyointestinalis*, two (13%) *C. coli*, and three (20%) *C. jejuni* strains were isolated from 15 *M. musculus* caught on a Dutch organic pig farm (Meerburg et al., 2006). *Campylobacter* spp. were, however, not isolated from the pig manure collected at the same farm. In the same study, on two other farms *C. coli* was isolated from 1/6 (16.7%) and *Campylobacter* spp. from 1/30 (3.3%) of *M. musculus*, respectively.

Concerning *S. araneus*, our results were in line with a previous study in which all shrews (10 *S. araneus* common shrews and 119 *Crocidura russula* greater white-toothed shrews), were negative for *Campylobacter* species (Meerburg et al., 2006). Another study, however, reported isolation of *C. jejuni* from the spleens of one water (*Neomys fodiens*) and one common shrew (*S. araneus*), even though they were not isolated from the gut, in the United Kingdom (Healing and Greenwood, 1991). Thus, it is also possible that the spleens of the animals collected in our study would have been positive for *Campylobacter*, but this was not tested.

*Rattus norvegicus* are widespread throughout most of the world and have viable populations in Finland. They are especially adapted to living in close proximity to human habitation and they may cause large economic losses by destroying materials, eating and defecating on food and feed, and by spreading disease. In this study, *C. jejuni* was identified in 20% (2/10) of the pooled *R. norvegicus* samples from farms. Previous studies have also shown that *R. norvegicus* may be carriers of *Campylobacter*. *C. jejuni* was isolated from three and *C. coli* from nine percent of *R. norvegicus*, respectively, collected from pig farms, chicken farms, and non-farm locations in Sweden (Backhans et al., 2013). In France, *C. jejuni* was identified from 40% of *R. norvegicus* collected from pig farms (LeMoine et al., 1987). Another study identified *C. coli* from 12.5% of *R. norvegicus* caught on a pig farm in Netherlands (Meerburg et al., 2006).

### Genotyping

For WGS, we chose farm isolates that represented all the different animal species in each sampling and, whenever possible, animals caught inside or in close proximity to production or storage buildings. In addition, all isolates from animals trapped in natural habitats were included for WGS analysis. The MLST types that occurred among the *Campylobacter* isolates differed mostly from the ones previously described from livestock and humans, and the majority of the STs were novel. A previously defined ST was available for only seven *Campylobacter* isolates out of all the 147 typed isolates in our study. Markedly, the majority (5/7) of these were isolated from on-farm pooled samples. These STs have previously been isolated from, e.g., wild birds in Sweden and New Zealand (ST 1304, ST-1304 CC), chicken offal or meat in Denmark (ST 4791, ST-45 CC), and various sources including chicken, cattle, dog, and human gastroenteritis worldwide (ST 2219, ST-45 CC). ST 2219 was isolated from *A. flavicollis* (one strain isolated from a pig farm sample), and the other STs from *R. norvegicus*, *M. glareolus*, and *A. flavicollis* from on-farm samples. Our findings are in agreement with previous studies finding also similar genotypes from small mammals and livestock (Adhikari et al., 2004; Williams et al., 2010), underlining the need for stringent biosecurity measures on farms. Unfortunately, food-producing animals were not sampled for *Campylobacter* in our study and thus we were unable to test the hypothesis that the *C. jejuni* isolates having ST-45 CC or other known STs originated from the farm animals. Furthermore, only one colony per pooled sample was analyzed and it is possible that an even higher proportion of livestock-associated STs might have been detected if several colonies were picked.

To our knowledge, MLST or WGS has only previously been used for typing *Campylobacter* isolates from small mammals in a South Korean study investigating isolates from *M. minutus* trapped in sesame fields (Kim et al., 2020), and in a United Kingdom study targeting *M. glareolus* and *A. sylvaticus* caught from a woodland habitat and six cattle farms (Williams et al., 2010). Our isolates, including those from *M. minutus*, differed in all seven loci of the MLST scheme from those reported from South Korea. The *Campylobacter* strains from the Finnish *M. minutus* trapped on-farm and from natural habitats in different locations, however, shared the same ST and the strains were very clonal, suggesting recent clonal expansion of the population in Finland. Williams et al. (2010) reported a single clonal *C. jejuni* population from *M. glareolus* with all the isolates representing the same ST 3704 regardless of whether they were caught from woodland or on farms. In contrast, we found only one *C. jejuni* having this ST from *M. glareolus* trapped in the woods while the remaining isolates had other, mainly novel sequence types. This finding most likely reflects geographical and temporal differences in the *M. glareolus* populations and expands our knowledge of this species as a carrier of different *C. jejuni* strains. However, we also found the same STs regardless of the trapping site (woods, field, or farm), further suggesting *C. jejuni* lineages specific to *M. glareolus*.

Concerning *R. norvegicus*, the Dutch study identified *C. coli* from 1/8 (12.5%) *R. norvegicus* caught on a pig farm in Netherlands (Meerburg et al., 2006). Amplified fragment length polymorphism (AFLP) typing, however, showed that the genotype differed significantly from that isolated from pig manure on the same farm (Meerburg et al., 2006). In another study, 86.7% of rats collected at a duck farm were *Campylobacter* positive (Kasrazadeh and Genigeorgis, 1987). The authors concluded that the most probable source of colonization of the ducks by *C. jejuni* were the rats and mice found in abundance on the premises, since rat and mice droppings were found in the duck feeding and watering troughs in that study. In our study, however, very few rats were caught, which is likely due to their neophobic behavior and the short sampling period on the farms. Thus, we could not thoroughly evaluate their role as a reservoir for *Campylobacter*. Our results, however, suggest that *R. norvegicus* may carry STs also identified in livestock, making them a possible vector of *Campylobacter* on farms. Since rats are prevalent in urban and rural settings, live in sewers and are in contact with human and livestock wastes, they would be an interesting subject for further studies.

cgMLST clustering showed that the strains from mice and voles were quite separate from the rest of the *C. jejuni* population (over 600 allele difference). The majority of the cgMLST alleles were segregated according to species (*A. flavicollis* and *M. minutus*) or group (vole). Very few or no alleles were shared with livestock or human *Campylobacter* strains. Core-genome phylogeny of the animal-associated strains confirmed the separation of the lineages as observed in cgMLST. The strains from voles formed a distinct clade that was clearly separate from *C. jejuni*, while the strains isolated from mice grouped monophyletically within the diversity of the *C. jejuni* population. Moreover, there was no difference between *Campylobacter* strains from farm versus wild captured animals, whose phylogeny followed the animal species-association indicating adaptation to different host species. The only major exceptions to this were the clustering of different *Campylobacter* strains from *M. glareolus*, *M. agrestis*, and *M. mystacinus* (syn. *M. levis*) isolated from different locations throughout Finland (both on-farm and natural habitats), and *M. glareolus* and *M. rutilus* isolated from northern Finland. The overlap in habitat selection among vole species and the commonness of *M. glareolus* could explain the spread of *Campylobacter* from *M. glareolus* to other sympatric vole species. The larger cluster of vole strains also contained the bank vole having ST 3704, the genotype that was previously also identified in the United Kingdom (Williams et al., 2010). The smaller cluster of isolates from voles was, however, particularly associated with *M. rutilus* in northern parts of Finland and seems more likely to have spread from this closely-related species to *M. glareolus*. The new strain/variant of campylobacter found in the red vole, *M. rutilus*, is interesting since this vole species has a wide northern distribution in the Holarctic; from northernmost Fennoscandia over north Russia and Siberia to northern North America, which may suggest that this strain could be widely spread in northern regions.

FastANI further indicated that the vole strains might form a different species or sub-species of *Campylobacter*. Usually a species is defined to include strains that share ≥95% ANI (Jain et al., 2018). However, *C. coli* is known to form lineages (clades 1, 2, and 3) that are almost as separate as different species. The ANI value for *C. coli* clade 3 versus clade 1, which included most of the human and farm animal isolates (Sheppard et al., 2013; Skarp-de Haan et al., 2014), for example, is ∼92%. The *Campylobacter* strains from voles showed 90 to 91% ANI with *C. jejuni* and <85% ANI with *C. coli* (same with *C. hepaticus*), while strains from mice shared ≥95% ANI with both *C. jejuni* subspecies, clearly indicating that the mice strains belong to *C. jejuni*, more specifically *C. jejuni* subsp. *jejuni*. The isolates from voles tested hippurate positive in the phenotypic hippurate hydrolysis test. However, the majority of them were only weakly identified as *C. jejuni* using MALDI-TOF-MS, further suggesting they may represent a novel sub-species.

### Antimicrobial Resistance Markers

Clinically relevant resistant markers were not found in our study. The only known mutation that was identified was that leading to the amino acid change P104S in the Gyrase subunit A, which has been linked to fluoroquinolone resistance only in connection with other mutations in *gyrA* (Hakanen et al., 2002; Piddock et al., 2003; Payot et al., 2006). It is therefore unlikely that the isolates in this study would be resistant to quinolones. Additionally, slightly more than one-third of the isolates harbored a beta-lactamase gene. This proportion is lower compared to some previous studies that report even 80–90% of *C. jejuni* strains isolated from various sources producing beta-lactamases and/or harboring beta-lactamase genes (Lachance et al., 1991; Tajada et al., 1996; Marotta et al., 2019). Although the presence of beta-lactamases has been shown to lower the MICs for certain beta-lactams in *Campylobacter* spp., campylobacters are generally considered intrinsically resistant to many agents in this group and beta-lactams are not recommended in the treatment of campylobacteriosis (Aarestrup and Engberg, 2001; Griggs et al., 2009; Wieczorek and Osek, 2013). The type of resistance marker present in the isolates was linked with the ST, with all the isolates of the same ST harboring identical markers. This likely reflects the clonality of the isolates, rather than any external selective pressure.

### Conclusion

Rodents, especially *A. flavicollis* and *M. glareolus*, were frequent carriers of *Campylobacter*. However, the majority of the detected genotypes differed markedly from those circulating in humans and livestock. Occasionally rodents, especially the species living in close connection to humans, may also carry strains associated with colonization in livestock and disease in humans. How transient the colonization is, remains an open question. It is possible that rodents participate in the maintenance of bacteria on farms, even though they are not the original source of the *Campylobacter* strains circulating in livestock. Further studies should be conducted to confirm this.

## Data Availability Statement

The raw sequence data produced in this study was submitted to the European Nucleotide Archive (ENA) database and is publicly available under the project accession number PRJEB37870.

## Ethics Statement

Animal trapping was carried out according to stipulations of national animal welfare and environmental legislature (permits from Ministry of Agriculture and Forestry of Finland and Ministry of the Environment of Finland). The Animal Ethics Council of the State Provincial Office of Southern Finland has prior to this study ruled that terminal snap trapping of small rodents does not constitute an animal experiment and therefore does not require an animal experimentation permit. No human studies are presented in this manuscript. No potentially identifiable human images or data is presented in this study.

## Author Contributions

RK, MR, MH, PT, MS, OH, SO, JN, and HH contributed to the conception and design of the study. JN, OH, and HH collected and prepared all the animal samples. MR, JT, MS, AJ, SO, and RK analyzed the data. MR performed the phylogenomic analyses. MR and RK interpreted the results. RK performed the statistical analysis. MR wrote the first draft of the manuscript. RK, SO, and MS wrote sections of the manuscript. All authors made substantial intellectual contributions, revised the manuscript critically, and approved the final version of the manuscript.

## Disclaimer

MR is employed with the European Food Safety Authority (EFSA) in the Unit BIOCONTAM that provides scientific and administrative support to EFSA’s Scientific Activities in the area of Rapid Outbreak Assessment and molecular typing of foodborne pathogens. However, the present article is published under the sole responsibility of MR and may not be considered as an EFSA scientific output. The positions and opinions presented in this article are those of the author/s alone and are not intended to represent the views/any official position or scientific works of EFSA. To know about the views or scientific outputs of EFSA, please consult its website under http://www.efsa.europa.eu.

## Conflict of Interest

The authors declare that the research was conducted in the absence of any commercial or financial relationships that could be construed as a potential conflict of interest.

## Footnotes

1
https://hub.docker.com/r/ummidock/innuca:3.2

2
https://www.bioinformatics.babraham.ac.uk/projects/fastqc/

3
https://pubmlst.org/

4
https://hub.docker.com/r/mickaelsilva/chewbbaca_py3

5
http://doi.org/10.5281/zenodo.1322564

6
https://github.com/cimendes/proCompare

7
https://hub.docker.com/r/ummidock/prokka

8
https://hub.docker.com/r/sangerpathogens/roary

9
https://www.ebi.ac.uk/Tools/msa/clustalo/

10
https://blast.ncbi.nlm.nih.gov/Blast.cgi

## Supplementary Material

Supplementary Dataset 1 Assembly statistics.

Supplementary Dataset 2 Metadata used in the cgMLST analysis including year of isolation, country, 7-gene MLST sequence type, and source for all strains.

Supplementary Dataset 3 goeBURST groups based on the cgMLST schema (678 loci), at different thresholds of shared loci.

Supplementary Dataset 4 Allele segregation analysis showing shared loci according to source calculated using the cgMLST profiles of 4,323 strains for which source information was available (Supplementary Dataset 2).
