# Correction: Host-dependent clustering of *Campylobacter* strains from small mammals in Finland

**DOI:** 10.3389/fmicb.2025.1656693

**Published:** 2025-08-04

**Authors:** Frontiers Production Office

**Affiliations:** Frontiers Media SA, Lausanne, Switzerland

**Keywords:** *Campylobacter jejuni*, phylogeny, comparative genomics, rodent, mouse, vole, shrew

The Data availability statement was erroneously given as “The raw sequence data produced in this study was submitted to the European Nucleotide Archive (ENA) database and is publicly available under the project accession number PRJEB28441.”

The correct Data availability statement is “The raw sequence data produced in this study was submitted to the European Nucleotide Archive (ENA) database and is publicly available under the project accession number PRJEB37870.”

The original version of this article has been updated.

